# VA Vascular Injury Study (VAVIS): VA-DoD extremity injury outcomes collaboration

**DOI:** 10.1186/1471-2482-15-13

**Published:** 2015-02-03

**Authors:** Paula K Shireman, Todd E Rasmussen, Carlos A Jaramillo, Mary Jo Pugh

**Affiliations:** Department of Surgery, Sam and Ann Barshop Institute for Longevity and Aging Studies, University of Texas Health Science Center San Antonio, The South Texas Veterans Health Care System, 7703 Floyd Curl Drive, MC 7790, San Antonio, TX 78229-3900 USA; US Combat Casualty Care Research Program, Fort Detrick, Frederick, MD 21702-5012 USA; The Uniformed Services University, Bethesda, MD 20814 USA; Department of Rehabilitation Medicine, University of Texas Health Science Center San Antonio, Polytrauma Rehabilitation Center, South Texas Veterans Healthcare System, 7400 Merton Minter BLVD, San Antonio, TX 78229 USA; Department of Epidemiology and Biostatistics, University of Texas Health Science Center San Antonio, The South Texas Veterans Health Care System, 7400 Merton Minter BLVD, San Antonio, TX 78229 USA

**Keywords:** Extremity vascular injury, Limb salvage, Outcomes, Transitions of care, Service members, Veterans, Iraq, Afghanistan

## Abstract

**Background:**

Limb injuries comprise 50-60% of U.S. Service member’s casualties of wars in Afghanistan and Iraq. Combat-related vascular injuries are present in 12% of this cohort, a rate 5 times higher than in prior wars. Improvements in medical and surgical trauma care, including initial in-theatre limb salvage approaches (IILS) have resulted in improved survival and fewer amputations, however, the long-term outcomes such as morbidity, functional decline, and risk for late amputation of salvaged limbs using current process of care have not been studied. The long-term care of these injured warfighters poses a significant challenge to the Department of Defense (DoD) and Department of Veterans Affairs (VA).

**Methods/Design:**

The VA Vascular Injury Study (VAVIS): VA-DoD Extremity Injury Outcomes Collaborative, funded by the VA, Health Services Research and Development Service, is a longitudinal cohort study of Veterans with vascular extremity injuries. Enrollment will begin April, 2015 and continue for 3 years. Individuals with a validated extremity vascular injury in the Department of Defense Trauma Registry will be contacted and will complete a set of validated demographic, social, behavioral, and functional status measures during interview and online/ mailed survey. Primary outcome measures will: 1) Compare injury, demographic and geospatial characteristics of patients with IILS and identify late vascular surgery related limb complications and health care utilization in Veterans receiving VA vs. non-VA care, 2) Characterize the preventive services received by individuals with vascular repair and related outcomes, and 3) Describe patient-reported functional outcomes in Veterans with traumatic vascular limb injuries.

**Discussion:**

This study will provide key information about the current process of care for Active Duty Service members and Veterans with polytrauma/vascular injuries at risk for persistent morbidity and late amputation. The results of this study will be the first step for clinicians in VA and military settings to generate evidence-based treatment and care approaches to these injuries. It will identify areas where rehabilitation medicine and vascular specialty care or telehealth options are needed to allow for better planning, resource utilization, and improved DoD-to-VA care transitions.

## Background

### Combat-related vascular injuries

Wartime injuries have been studied during every major conflict of the 20^th^ and 21^st^ centuries. Hughes in Korea [1], and Rich in Vietnam [2], each advanced the care of Service members with vascular injuries. Ligation of injured blood vessels and the resultant high limb loss rate in World War II gave way to arterial and venous repair in the Korean and Vietnam Wars. With these advances, life and limb salvage rates improved dramatically in the short and mid-term time frame. However, long term outcomes following the definitive repair of wartime vascular injuries by a vascular specialist in the theater of war have not previously been evaluated. *To improve care, we must provide long term follow up and define the natural history of wartime vascular injury over an injured Soldier’s lifetime.*

Improvements in battlefield protection devices, casualty evacuation and medical care have enhanced survival rates, currently over 90%, following combat injuries. Many survivors of once-lethal injuries now require prolonged medical and rehabilitation care for polytrauma injuries [3]. Rapid aero-medical evacuation from the battlefield to definitive surgical care has been a major contributor to improved initial limb salvage rates, allowing access to vascular surgery care more rapidly, thereby decreasing ischemia times of injured limbs. The in-theatre use of diagnostic angiography and endovascular treatments has enabled more attempts at limb salvage after extremity vascular injury [4]. Additionally, patients usually leave the theater of war within 24 hours after receiving initial medical treatment [4–6]. Subsequent care is obtained in several locations while en route to the United States, and in military trauma centers in the U.S. Once the acute hospitalization phase is completed, and longer term rehabilitation at Walter Reed National Military Medical Center or the San Antonio Military Medical Center is completed, the patient will be released to the home station and the associated military treatment facility (MTF) or to the local community. In the event that the Service member separates from the DoD, care is often transferred to the Veterans Administration (VA), especially when high service-connected ratings of disability are present. Most of the MTFs do not have a vascular surgeon or non-invasive vascular laboratory (duplex ultrasound) to provide long-term surveillance of the vascular repair.

### Prospective and retrospective studies of orthopaedic and vascular injury are limited

The Lower Extremity Assessment Project (LEAP) was a prospective, longitudinal study performed at 8 level 1 civilian trauma centers to study the outcomes of lower extremity orthopaedic trauma to answer the questions surrounding the decision to amputate or salvage injured limbs [7]. While there were no controls for the therapeutic interventions, long-term follow up of 7 years is available on most patients. The results of multiple studies emanating from this trial are reviewed in Higgins *et al*. [7] Major findings include similar self-reported health status measures 2 years after injury in patients undergoing amputation compared to limb reconstruction. However, patients with limb reconstruction had a higher rate of complications and rehospitalization [8]. While two year health care costs were similar between patients with limb salvage and amputation, prosthesis-related expenses increased the costs for patients with amputations and the projected lifetime health care costs were three times higher for patients with amputation compared to limb salvage [9]. None of the lower extremity injury severity indices have been shown to be sensitive predictors of which patients should undergo initial amputation versus limb reconstruction [7]. The only study that focused on vascular injuries was in association with knee dislocation; this study contained 18 patients and suggested that outcomes in revascularized patients were better than those undergoing amputation [10].

The Military Extremity Trauma Amputation/Limb Salvage (METALS) study applied methods similar to those in the LEAP study to the Operation Enduring Freedom/Operation Iraqi Freedom/Operation New Dawn (OEF/OIF/OND) military population [11]. This retrospective cross-sectional study included 324 U.S. Service members who had sustained major lower limb trauma as a result of high-energy blast and ordnance-related mechanisms. Both unilateral and bilateral lower limb traumatic injuries were included. Clinical data were obtained from participants’ medical records and through telephone interviews. Patients in the limb salvage and amputation groups reported significant disability compared to normal populations and 38% screened positive for depressive symptoms with 34% not working or in school at the time of the interview. Of interest, amputees reported better scores on the SMFA compared to limb reconstruction patients as well as lower rates of post-traumatic stress disorder. Only 17 limb salvage patients (~10%) in the study underwent revascularization [11]. These findings are in contrast to LEAP which reported similar health status measures in amputees and limb salvage patients, suggesting that translation of findings from non-combat civilian settings to combat injury may not be generalizable.

The Late Amputation Study Team (LAST) was to determine the prevalence of late amputations in wounded Service members from OEF/OIF/OND. While most amputations occurred in the acute setting, 15% of Service members sustaining lower extremity injuries had late amputations, defined as greater than 12 weeks after injury, range 12 weeks to 5.5 years [12]. A subset of these patients with type III open diaphyseal tibia fractures managed with late amputation were shown to have an increased incidence of infection [13].

### Studies examining long term outcomes associated with vascular interventions are limited

In the Global War on Terror Vascular Initiative (GWOT VI) Rasmussen and colleagues defined the patterns and types of injuries [5, 14] as well as short term outcomes of using temporary vascular shunts [6], efficacy of incorporating endovascular procedures [4], and selective repair of tibial artery injuries [15]. OEF/OIF/OND vascular trauma occurred at a 5-times higher rate than previous wars and was caused predominantly by explosive (73%) and gunshot (27%) injuries [5]. The increased vascular trauma rate may be attributable to improvements in body armor, and increased survivability due to the use of tourniquets and shorter casualty evacuation times [5]. As a follow up to the acute care studies, the DoD team initiated a survey study using the SF-36®, SMFA and a questionnaire to determine the functional outcomes of Service members with extremity vascular injury repairs [16]. Long-term outcomes assessment for this study was limited by the inability of the DoD team to access VA databases.

Similar to military trauma, multiple reports document the acute care of patients with civilian vascular extremity injuries, but limited data on small sample sizes is available for long-term outcomes [17]. Thirteen of 22 patients were available with a mean follow-up of 59 months demonstrating graft patency by duplex ultrasound [17]. Additional studies [18–20] on small numbers of patients with long term follow up used ankle-brachial indices or clinical evaluation, which are often inaccurate methods to assess graft patency [21].

What we know about long-term treatment for patients following vascular repair is based on vascular repair due to atherosclerotic disease. Evidence-based treatments recommended in this patient population include antiplatelet therapy and *duplex ultrasound graft surveillance.* Antiplatelet therapy, especially aspirin, is considered standard of care for patients with atherosclerotic disease to prevent death and disability from stroke and myocardial infarction. Aspirin doses of 80 to 325 mg/day are effective compared to higher-dose aspirin which can increase side effects [22]. Multiple studies and reviews have concentrated on drug therapies to increase patency rates in extremity vascular bypasses, all focusing on patients with atherosclerosis. The Antiplatelet Trialists’ Collaboration found that antiplatelet therapy was associated with a relative risk reduction of 43% in graft occlusion [23]. However, the optimal therapies that maximize graft patency and minimize complications may vary depending on a variety of factors, including whether a venous or prosthetic graft is used. The Dutch Bypass Oral Anticoagulant study (BOA) compared vitamin K antagonists to aspirin and determined that vitamin K antagonists were more beneficial in patients with vein grafts, albeit with a higher rate of major bleeding episodes, while aspirin improved patency in prosthetic grafts [24]. Possible explanations for these observations include that aspirin may decrease platelet deposition on the more thrombogenic surface of prosthetic grafts while the coagulation cascade may be more critical in vein graft occlusions [25]. Despite the BOA study, the Seventh American College of Chest Physicians Conference on Antithrombotic and Thrombolytic Therapy evidence-based guidelines recommended using aspirin, rather than vitamin K antagonists, in both venous and prosthetic bypass grafts and the combination of aspirin plus vitamin K antagonists for patients with a high risk of graft thrombosis and limb loss, noting that the recommendations placed a high value on the avoidance of bleeding complications [22]. A Cochrane review for preventing thrombosis after arterial surgery concluded that limited evidence suggested antiplatelet agents such as aspirin, pentoxifylline, or aspirin given with dipyridamol improved graft patency compared to no treatment. However, patients with prosthetic grafts benefited more from aspirin or aspirin given with dipyridamol than patients with venous grafts. For venous grafts, vitamin K antagonists may be more beneficial and a combination of aspirin and clopidogrel might be as effective as vitamin K antagonists [26]. These observations led to the Clopidogrel and Acetylsalicylic acid in bypass Surgery for Peripheral Artery disease (CASPAR) trial comparing aspirin plus clopidogrel to aspirin alone in patients undergoing below the knee bypasses for peripheral arterial disease. In the overall population, graft patency was similar in patients receiving aspirin plus clopidogrel compared to aspirin alone. However, subgroup analysis revealed an increased graft patency for aspirin plus clopidogrel in patients receiving prosthetic grafts without increasing major bleeding complications [25]. Finally, a systematic review of endovascular therapies found evidence to support the recommendation of aspirin 50 to 300 mg with clopidogrel possibly being an alternate therapy [27].

Statin use has been associated with increased cardiovascular graft patency, see [28] for recent review. While statins were associated with improved 1 year survival in patients receiving lower extremity grafts for atherosclerotic disease, graft patency was not affected by statin use [29]. Statin use and graft patency remains an open critical question; especially given adverse effects (e.g. myopathy) associated with statin use [30].

*Duplex ultrasound graft surveillance* is the standard of care for monitoring bypass grafts and vascular interventions including vein grafts, angioplasties and/or stents [21, 31–34]. This technique allows the physician to visualize the structure of the blood vessels, how blood is flowing through the vessels and measures the speed of the flow of blood. Duplex ultrasound can also be useful to estimate the diameter of a blood vessel/graft as well as determine if a stenosis/occlusion has occurred. This application has been extensively studied in patients with atherosclerosis as the etiology for requiring intervention [21, 31–34]. However, support for use of duplex ultrasound after vascular interventions performed following traumatic injury is limited [17]. *No information is available regarding the extent to which this standard of care is met either in the VA or DoD for vascular repairs performed in trauma patients.*

In summary, antiplatelet agents are a mainstay in the treatment of patients after bypass and endovascular therapies for atherosclerotic disease secondary to their salutary role in decreasing cardiovascular events and maintaining graft/endovascular repair patency. Exclusion criteria for randomized control trials often exclude patients with risk factors for bleeding; thus, the incidence of complications in the general population with these treatments may be different than in the trials. Statin use has been advocated to improve vascular graft patency; however, prescription patterns in Veterans with vascular repairs for trauma are unknown. Duplex ultrasound graft surveillance has also been used extensively in patients undergoing vascular repair for atherosclerosis to detect graft failure. Finally, studies in atherosclerotic patients may not translate to the predominately younger trauma population and the etiology of graft failure may be different in trauma patients. Thus, understanding treatment practices of prescribing antiplatelet agents and other anticoagulants, and the associated complications with these treatments, as well the role of duplex ultrasound, will be a first step in defining the care of Veterans and civilians with extremity vascular trauma.

### Aims of the VA Vascular Injury Study (VAVIS)

**Figure 1:**
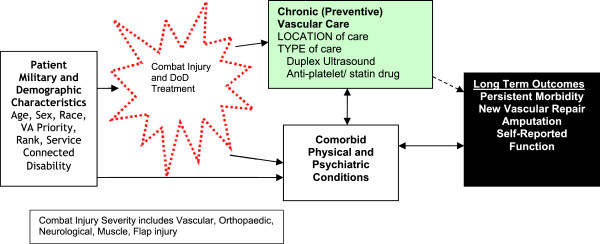
**Donabedian’s model of quality of care posits that processes of care (Vascular Preventive Care) may be associated with patient outcomes.** However, the complex relationships among demographic characteristics, initial injury severity, and comorbid physical and psychiatric conditions require careful attention to the potential for confounding and effect modifiers. While it is also important to control for demographic characteristics, particular concern is focused on controlling for disease burden and initial injury characteristics since these may have substantial impact on the outcome that is unrelated to the initial vascular injury and preventive care.

VAVIS is a longitudinal cohort study designed to examine outcomes associated with in-theatre vascular injury, funded by the Department of Veterans Affairs (VA), Health Services Research and Development Service. Figure 1 shows the theoretical model that guides our research. This model posits that understanding the health care delivery process for these injuries will result in a better understanding of their outcomes. Therefore, the goal of VAVIS is to characterize patients with initial in-theatre limb salvage (IILS) and identify late vascular surgery related limb complications in Veterans receiving VA vs. non-VA care.

Because little is known about the natural history of these types of injuries given modern combat casualty care, our first aim is to describe the population of interest including initial and subsequent treatment in the DoD. Our second aim is to describe extant preventive care processes provided for individuals with IILS by the VA since best practices for individuals with traumatic vascular repair have not been established and little is known about the types of preventive care available. Because research in other types of care have found that processes of care identified as best practices are associated with patient outcomes [35], our third aim is to identify the association of Vascular Preventive Care with patient outcomes [36] such as measures of persistent morbidity, adjusting for potential confounders, and effect modifiers. While it is also important to control for demographic characteristics, particular concern will be focused on controlling for traumatic brain injury, physical and psychiatric disease burden, and initial injury characteristics since these may have substantial impact on the outcome that is unrelated to the initial vascular injury and preventive care [37]. If Vascular Preventive Care processes are associated with positive patient outcomes, there will be support for these care processes as best practices [38].

Our fourth aim is to describe long-term patient-reported outcomes in Veterans with traumatic vascular limb injuries controlling for potential confounders such as complexity of the combat injury (i.e., involvement of neurological and musculoskeletal components), traumatic brain injury, and comorbid mental and physical health conditions. Improvements in battlefield care, such as the use of temporary vascular shunts, transfusion methods, *etc.*, have evolved at a very rapid pace, but there is a need to understand the long-term outcomes of these treatments to inform clinical decision making and thus fully optimize trauma systems fielded in-theater. Findings from VAVIS will provide insight for military trauma, vascular and orthopedic surgeons to know which types of injuries can be repaired with adequate functional outcome to justify the initial surgical decision. It can be a particularly traumatic and demoralizing experience for a patient to undergo multiple procedures to attempt limb salvage only to lose the limb later due to ischemia, infection, extensive musculoskeletal injury or pain. A better understanding of outcomes will identify which patients may benefit from initial amputation versus attempts at vascular repair.

## Methods/Design

This observational cohort study of Veterans who receive VA care for IILS will use retrospective data from the DoD and VA that was collected for the GWOT VI study (DoD) and the Trajectories of Resilience and Complex Comorbidity (TRACC [VA]) studies in addition to interview and survey data collected during the course of this study.

### Study sample

Our denominator includes all individuals verified as having undergone initial in-theatre limb salvage (IILS) during the GWOT VI study. Inclusion criteria for IILS included: 1) received a vascular repair on the affected limb(s) and 2) transferred to the post-anesthesia unit and to the ICU after surgery. Individuals with amputations performed as the first, in-theatre operation are excluded.

**Figure 2:**
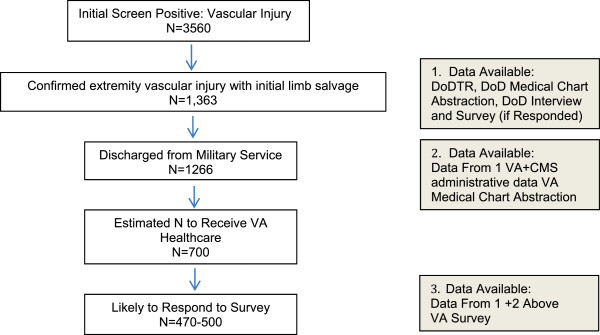
**Eligibility flow diagram showing DoD chart abstractions using the Department of Defense Trauma Registry (DoDTR).**

To date, DoD chart abstractions using the Department of Defense Trauma Registry (DoDTR) have identified 1,363 individuals with confirmed extremity vascular injury and initial limb salvage; approximately 1,266 of these have separated from active service (Figure 2). Based on VA enrollment data, approximately 59% of OEF/OIF/OND Veterans discharged from the military have received VA health care [39], we expect approximately 700 individuals in the VA denominator. Database and chart abstraction components will include all those who received VA care (estimated N = 700). Additional analyses will be conducted for those who do not receive VA care within limits of available data.

### Study procedures

The VAVIS protocol was reviewed and approved by the VA Central IRB. This three component study will begin first by compiling data previously collected from DoD and VA (FY01-FY14), linking files from each source using the unique identifier. Once VA patients in the confirmed IILS cohort are identified, we will conduct medical chart abstraction of VA electronic medical records, and conduct a patient interview and survey to obtain patient reported outcomes. The study will start on April 1, 2015 and will be open for three years.

### Previously collected data

Data abstracted from the DoD medical chart in the GWOT VI study will be used in this study as control variables, or characteristics for stratification based on expert consensus of our clinical team. Table 1 provides a description of the type of information included in the GWOT VI dataset. These DoD data will be merged with national VA health care system data collected as part of the TRACC study (VA inpatient, outpatient, pharmacy and non-VA care [Fee Basis/ Medicare] data). Table 2 provides a description of the variables, definitions and data sources used to create variables. For those who have received VA or Medicare care, we will develop variables related to indicators of persistent morbidity, type of care received, and long-term outcomes. Table 3 provides a specific description of these measures and how they are defined.

**Table 1:** **Information obtained from DoD chart abstraction: baseline polytrauma injury characteristics**

Type of variable	Information
Demographics	Age, sex, race/ethnicity and service branch
Initial Injury Information	Date, primary cause/mechanism
Vascular Extremity Details	Arterial/venous injury location, pre/post repair details, and complications.
Ortho/Nerve Extremity Details	Associated soft tissue/bone/nerve injuries, methods of repair, flap status.
Follow Up Care	Dates, location, and CT/MRI/Angio/Duplex studies.
Functional/ Amputation status	Limitations, context of amputation if relevant

**Table 2:** **Data variables, definitions, and data sources**

Variables	How defined	Data source
**Demographic Characteristics**	Age, Sex, Race/ Ethnicity, Education	OEF/OIF/OND Roster File (from Environmental Epidemiology Service)
**Military Characteristics**	Rank, Component (active, guard, reserve), Service Branch,	
**Military Discharge Date**	Military discharge dates	
**Other Demographic Characteristics**	Marital status	National VA Inpatient and Outpatient Care Files (Medical SAS data sets)
**Diagnoses** for which care is received	ICD-9-CM Codes each visit	
**Location of Care/ Facility Type** (e.g. Medical Center, outpatient clinic, community based outpatient clinic)	STA5A/STA6A; Last letter of code indicates the type of facility	
**Type of care**: Relevant specialty care: vascular surgery clinic (415), vascular lab (421), amputation clinic (418), prosthetic clinic (417), PM&R (201,211).	DSS Identifier (stop code)	
**Procedures:** tests such as duplex ultrasound or type of surgery when admission is on day of surgery	CPT-4 Codes	
**Geospatial data** of patient home to identify distance to primary station and clustering of these Veterans across the country.	Zip Code, Longitude/latitude of patient residence	
**Diagnoses** for which care is received	ICD-9-CM Codes each visit	National VA Inpatient Care Files (Medical SAS data sets from national repository at the Austin Automation Center)
**Location of Care/ Facility Type** (e.g. Medical Center, domiciliary, neuro/psychiatric hospital)	STA5A	
**Provider Specialty**: Bedsection is mapped 1:1 with provider specialty (e.g., vascular [106] and peripheral vascular [62].	Bedsection	
**Procedures:** tests such as duplex ultrasound or type of surgery	ICD-9A Codes	
**Geospatial** (same as above)	Zip Code, Longitude/latitude patient home	
**Medications Prescribed** (e.g., opioids, other medications for pain, antibiotics for persistent infections such as osteomyelitis, antiplatelet agents, statins)	VA Product name, strength, day supply, number of pills dispensed Medicare Part D National Drug Code (NDC)	National Pharmacy Files from the Pharmacy Benefits Management Strategic health Group, Medicare Part D
**Dose** of medications average monthly dose (dose per unit × day supply)	Generic, day supply, number of units, dose, Medicare Part D: NDC code	
**Duration** of use	Day supply summed over time	
Specific Prosthetic Device	HCPCS PSAS, HCPCS CPT codes identify the specific item	National Prosthetics Patient Database (NPPD)
Specific Service Connected Disability and % disability for each condition	VETSNET condition code, %, number of disabling condition, service connected disability indicator	Veterans Service Network (VETSNET) Corporate Mini Master File (VETSNET File) derived from the Veterans Benefits Administration (VBA) Corporate Database.
Pain Scores: longitudinally (1–10 each visit)	Pain score	Corporate Data Warehouse

**Table 3:** **Indicators of persistent morbidity**

Indicators of persistent morbidity	Data used to identify	Data source
Use of unscheduled or emergency care for vascular issues	DSS Clinic Identifier and ICD-9 Codes	Outpatient (OPC) files; VA-CMS
Use of scheduled care for polytrauma vascular issues	DSS Clinic Identifier and ICD-9 Codes	OPC files; VA-CMS
Long-term use/escalating dose of opioid medications	VA Product, Dose, Day Supply	Pharmacy Benefits Management (PBM) Data; Medicare Part D VA-CMS
	NDC Code	
Trajectory of chronic pain (latent class analysis of pain scores from TRACC)	Pain Score	CDW vitals
Conditions indicative of persistent morbidity (e.g., Venous stasis disease/venous ulcers, lymphedema)	ICD-9 Codes including but not limited to 729.81, 782.3, 729.5, 415.19, 451.11, 451.19 451.83, 451.82, V72.83	PTF, OPC files, VA-CMS
Prescriptions for compression stockings and wound care supplies (e.g., wound vac) indicative of swelling, venous stasis disease	VA Class	PBM Data, VA-CMS Durable Medical Equipment
	XA	
Use of prosthetic/orthotic devices (canes, walkers, special shoes, etc).	Health Care Financing Administration Coding System (HCPCS) Prosthetic and Sensory Aids Service (PSAS) Code;	National Prosthetics database
		CMS Durable Medical Equipment
Additional procedures (open or endovascular) on the injured extremity	CPT-4; ICD-9A procedure codes	OPC/PTF VA-CMS
Chronic antibody use (osteo or other potential infectious issues with the limb)	VA Product	PBM Data; Medicare Part D VA-CMS
Duplex ultrasound, MRA/CTA or angiogram to evaluate the repair	CPT-4 procedure codes including but not limited to 93970, 93971, 93975, 93976, 93978, 93979, 93922, 93923, 93925, 93926, 93930, 93931, 93924	OPC/PTF; VA-CMS
Mean pain scores over 4	Pain Score Mean per year	Corporate data warehouse Vital Signs
Proportion of VA care	Visits VA/ Total Visits	OPC/PTF; VA CMS outpatient/inpatient
Mortality	Date of Death	VA Vital Status file

### VA medical chart abstraction

We will access the electronic medical record for any VA patient with IILS from the time of VA entry to the time of abstraction, with special attention to functional outcomes including physical therapy, prosthetic care, and care for vascular injuries or conditions such as skin ulcers that may result from graft failure or complications of the initial injury. After development of the electronic data collection template, database, and training, the PI and the chart abstractors will independently review 10 charts and meet together to identify inconsistencies, errors, or disagreements and resolve any by consensus. This will continue until there is 90% agreement on all items abstracted.

### Survey data

After obtaining the appropriate IRB approvals, HIPAA, and OMB waivers we will proceed with the interview and survey component using a modification of the Dillman method [40]. During the chart abstraction process, we will obtain the contact address for individuals in the cohort and send a pre-notification letter, which will include a study information sheet, directions on how to access the survey online, and a toll-free telephone number to call to opt out of the survey. Two weeks later we will call individuals who have not opted out. Research staff will first obtain verbal consent from the Veteran to proceed and then will conduct the initial interview that addresses the Veteran’s understanding of their injury, vascular repair, and medical care since the original injury. The research staff will then answer questions regarding completion of the outcomes survey, which will be offered online or by mail/telephone options. If the Veteran requests a mailed survey, the research team will promptly send a packet containing the survey, and a stamped envelope for the participant to return the survey. If the individual requests telephone survey, research staff will offer the Veteran the opportunity to complete the survey at that time or to schedule a time convenient for the Veteran.

For the online/mail survey, one week after the interview, the research team will send a letter thanking those who have responded and remind non-respondents to reply, followed 2 weeks later by a second mailed invitation to non-respondents to complete the on-line survey (or mailed back-up). We will provide a gift card to participants who complete the mailed surveys.

The primary survey outcome measure for this study is one used for the GWOT VI: the *Short Musculoskeletal Function Assessment*. Additional instruments include the Paffenbarger Physical Activity Questionnaire, Chronic Pain Grade Scale, and the Military to Civilian Questionnaire MC2-Q (a measure of community re-integration). *Short Musculoskeletal Function Assessment* (SMFA) is a validated, 46-item self-reported health status questionnaire on extremity functional status. The SMFA contains two parts: the dysfunction index and the quality-of-life limitation index. The SMFA questionnaire has excellent internal consistency and stability, with most values greater than 0.90. Content validity for the dysfunction and bother indexes was supported with few ceiling effects (less than 5 percent), and no floor effects. Moreover, significant correlations were found between the SMFA dysfunction and other indexes and the physicians’ ratings of patient function (*e.g*., recreational and leisure activities, activities of daily living, and emotional function [rho ≥ 0.40]) and clinical measures including grip strength and walking speed [rho ≥ 0.40] [41].

*The Paffenbarger Physical Activity Questionnaire* [42] (PPAQ) is a validated scale that describes participation in physical activity more specific to sports and leisure which has been used in a number of prior studies including the METALS study [11]. This instrument asks respondents to identify up to 4 activities performed in the past three months, the amount of time spent in those activities, and the intensity of those activities. This instrument allows a calculation of the approximate metabolic equivalents (METS) expended (e.g. an adult sitting quietly expends 1 MET). We will use Ainsworth’s Compendium of Physical Activities [43] to classify reported activity by the rate of expended energy into METS in the same manner used by the METALS study to improve comparability to findings.

*The Chronic Pain Grade (CPG) scale* [44] is a validated measure of the severity of chronic pain. Similar to the METALS study, we will use the pain interference questions to distinguish the extent to which physical function and activities of daily living are affected by the pain [11].

*The SF-8™* is the first SF survey to be constructed on the basis of empirical studies linking each questionnaire item to a comprehensive “pool” of widely used questionnaire items, including but not limited to the SF-36®, proven to measure the same health concepts. Because the item “pool” for each of the eight health concepts was calibrated on the same metric as the corresponding SF-36® Health Survey scale, each SF-8™ single-item scale and the SF-8™ summary measures can be scored on the same norm-based metrics as the SF-36® scales and summary measures. Thus, the SF-8™ is an 8-item version of the SF-36® that yields a comparable 8-dimension health profile and comparable estimates of summary scores for the physical and mental components of health [45]. Therefore, we will use the SF-8™ rather than the SF-36™.

The Military to Civilian Questionnaire is a validated, 16 item self-reported measure of experiences during military to civilian transition, developed by Sayer and colleagues at the Minneapolis VA/Polytrauma Center [46]. It was validated using a, stratified sample of 1,226 Iraq and Afghanistan Veterans who used VA medical care. The internal consistency of the M2C-Q was .95 in this sample. Factor analyses indicated a single total score was the best-fitting model. Total scores were associated with measures theoretically related to reintegration difficulties including perception of overall difficulty readjusting back into civilian life (R^2^ = .49), probable PTSD, probable problem drug or alcohol use, and overall mental health. Subgroup analyses revealed a similar pattern of findings in those who screened negative for PTSD.

In addition to these outcome variables, we will collect self-reported behavioral health symptoms.

Depression Severity will be assessed with the Center for Epidemiologic Studies Short Depression Scale (CES-D 10), which has been demonstrated to be a valid and reliable measure to assess depression in both VA and non-VA settings [47–49]. The 10 items are rated on a 0 (rarely or none of the time) to 3 (all of the time) scale. Items for this instrument have not been directly mapped onto the 9 DSM IV diagnostic criteria for Major Depression, but the sensitivity and specificity average 80% and 70% respectively with regard to results from a formal diagnostic interview [47, 48]. PTSD Severity will be assessed with the PTSD Checklist-Military (PCL-M) [50]. The PTSD Checklist is a 17-item self-report measure designed to assess the diagnostic criteria of re-experiencing, avoidance and numbing, and hyperarousal. Respondents indicate on a 5-point scale (“Not at all” to “A great deal”) how much they were bothered by each symptom in the past month.

### Other data for spatial analysis

Research staff will contact vascular surgery, surgery, and physical medicine and rehabilitation clinics at each VA hospital to determine the extent to which VA facilities use contracts for vascular surgery patients and will identify sites with external contracts for vascular surgery, rehabilitation medicine and prosthetics care to create a data set to be used in our spatial analysis.

### Analytic plan

To address the first aim, we will compare injury, demographic and geospatial characteristics of patients IILS and identify late vascular surgery related limb complications and health care utilization in Veterans receiving VA vs. non-VA care using merged VA + VA-Medicare and DoD study data. We will identify vascular procedures performed, limb salvage survival time, and indicators of persistent morbidity over time (*e.g*., frequent/emergency care for vascular-related conditions, venous stasis disease, prosthetic use, long-term opioid therapy [VA patients only]). We will calculate descriptive statistics for all IILS patients’ demographic characteristics, long-term clinical outcomes (surgery related limb complications, time to amputation, persistent morbidity), and healthcare utilization (note that long-term use of opioid therapy will be assessed only among VA patients). We will also compare these descriptive statistics between those who received VA vs. non-VA care using chi-square tests for categorical variables and t-tests for continuous variables (with appropriate Box-Cox power transformation if necessary).

To address the second aim, characterize the preventive services received by individuals with vascular repair and related outcomes, we will first compute the proportion of patients who receive antiplatelet agents, statins, annual duplex ultrasound, and vascular specialty care (and categories described above), and the percent patency each year for individuals without amputation. We will then examine the relationship between percent patency and preventive care using random effects analyses that models the vascular repair patency as a linear function of the indicator of receiving antiplatelet agents, statins, duplex ultrasound [51, 52], and other covariates/confounders (e.g., number of limbs affected, any initial, early or late amputations, mental health comorbidity, TBI diagnosis, and pre-existing chronic disease states such as atherosclerosis, diabetes, peptic ulcer disease), where the random effect is used to adjust for the within facility correlation, and the inverse of the propensity score for receiving antiplatelet agents/statins/yearly duplex ultrasound is adjusted for as weights. The adjustment of the propensity score for receiving antiplatelet agents/statins/yearly duplex ultrasound will minimize the estimation biases due to confounding by indication. We will also create different cut points (e.g. 75%, 50% patent), and use a binary outcome model. If Vascular Preventive Care processes are associated with positive patient outcomes, there will be support for these care processes as best practices.

We will also conduct generalizability analysis using the inverse propensity probability weight technique. [53, 54] The goal is to examine whether the outcome model derived from the VA IILS patients (as described above) can be generalized to the non-VA IILS patients. We first derive model estimates for the VA cohort, then compute the propensity score for receiving non-VA care by modeling the log-odds of receiving non-VA care (vs. VA care) as a linear function of baseline covariates. The inverse propensity weighting is used to calibrate the outcome model derived from the VA IILS patients to the non-VA IILS patients. Finally we assess model generalizability by comparing model estimates between the VA vs. non-VA patients using the diagnostic method by Stuart et al. [55] This generalizability method does not require collecting outcomes for non-VA patients. However the credibility of this method relies on the “ignorability” assumption to hold true: conditioning on the covariates, the counterfactual outcomes of the non-VA patients (the patency outcomes should they receive VA care) would have been the same as the outcomes of the VA patients, and similarly, the counterfactual outcomes of the VA patients (the patency outcomes should they receive non-VA care) would have been the same as the outcomes of the non-VA patients.

Based on preliminary data from the TRACC study, we anticipate that the proportion of OEF/OIF/OND VA patients with ILLS will receive antiplatelet agents/statins/or yearly duplex ultrasound will be no greater than 44.7%. We also assume that the mean vascular repair patency falls between 50%-60%, and that the increased patency due to receiving antiplatelet agents/statins/or yearly duplex ultrasound is no less than 12% (conservatively). Under these conditions, the study will have ≥0.84 power to determine if Vascular Preventive Care processes are associated with positive patient outcomes.

To address the third aim (Describe patient-reported functional outcomes in Veterans with traumatic vascular limb injuries) we will begin by examining descriptive statistics associated with SMFA, PPFA, CPG scale, SF-8™, and M2C-Q). We will compare scores on these instrument among IILS patients with and without indicators of persistent morbidity, controlling for possible confounders associated with having indicators of persistent morbidity (from Aim 1) including CES-D, and PCL-M. We will then conduct separate generalized estimation equation analyses for each outcome, modeling the mean of each outcome as a linear function of the indicator of having one or more indicators of persistent morbidity and other covariates [52].

Secondary analyses will stratify individuals by initial injury severity type (e.g., vascular only, vascular + orthopaedic, vascular + orthopaedic + nerve, etc.) to further control for initial conditions that may be more difficult to control for in multivariable analyses. We will also examine the role of various prosthetic devices, including the extent to which the technology of the device, the number of different prosthetic devices per limb, etc. mediate the relationship between injury severity, persistent morbidity and functional outcomes. Furthermore, we will conduct generalizability analysis (described above) to examine whether the outcome model derived from the VA patients can be generalized to the non-VA patients.

Our power analysis for the third aim is based on the primary outcome: SMFA. Based on preliminary data, we assume that the proportion of patients with persistent morbidity falls between 10% and 50%. We also assume that the effect size associated with persistent morbidity on SMFA, PPFA, CPG scale, SF-8™, and M2C-Q no less than 0.435. Under those conditions, the study will have >0.80 power to identify a true difference in outcomes among those with and without persistent morbidity.

## Discussion

This study will provide key information about the current process of care for OEF/OIF/OND Veterans with polytrauma/vascular injuries at risk for persistent morbidity and late amputation. The results of this study will be the first step for clinicians in VA and military settings to generate evidence-based treatment and care approaches to these injuries. It will identify areas where rehabilitation medicine and vascular specialty care or telehealth options are needed allowing for better planning, resource utilization, and improved DoD-to-VA care transitions. Using the DoDTR to identify injured Service members and a DoD-VA research team to determine the long term outcome of traumatic vascular injury repairs provides an unprecedented opportunity to define current processes of care and improve the evidence base for further treatment. In addition, methods used in VAVIS can be applied to other health care problems encountered in combat casualty care to determine long term outcomes. These studies have the potential to improve battlefield care, transitions of care between the DoD and VA, as well as to determine care teams within the VA to optimally treat these complex patients.

## Abbreviations

ABI: Ankle-brachial index
ACCESS: A Microsoft Office data management program
AHLTA: The armed forces health longitudinal technology application
AHRQ: Agency for Healthcare Research and Quality
ArcGIS: Arc geographic information system
BIRLS: Beneficiary identification records locator subsystem
BOA: The Dutch bypass oral anticoagulant study
CAPRI: Compensation and pension record interchange
CASPAR: The Clopidogrel and acetylsalicylic acid in bypass surgery for peripheral artery disease trial
CDW: Corporate data warehouse
CES-D: Center for epidemiologic studies short depression scale
CNS: Central nervous system
Co-I: Co-investigator
CPT codes: Current procedural terminology codes
CT/MRI: Computed tomography scan/magnetic resonance Imaging
DHI: Deployment health initiative
DOD: Department of Defense
DoDTR: Department of Defense Trauma Registry
DSS Identifier: Decision support system patient identifier
EB: Empirical bayes
EMR: Electronic medical records
ESRI: Environmental systems research institute
EVI: Extremity vascular injury
FY: Fiscal year
GI: Gastro-intestinal
HCPCS: The healthcare common procedure coding system
HERC: The health economics resource center
HIPAA: The health insurance portability and accountability act
IRB: Internal review board
JPTA: Joint patient tracking application
JTTS: Joint Theater Trauma System
LAST: The late amputation study team
LEAP: The lower extremity assessment project
LOBO: Long-term outcomes in burned OEF/OIF veterans
MC2-Q: Military to civilian questionnaire
METALS: The military extremity trauma amputation/limb salvage
MOU: Memorandum of understanding
MRA/CTA: Magnetic resonance angiography and computed tomography angiography
MRSA: Methicillin-resistant staphylococcus aureus
MTF: Military treatment facility
NIH: National institute of health
NPPD: National prosthetics patient database
OEF: Operation Enduring Freedom
OIF: Operation Iraqi Freedom
OMB: Office of Management and Budget
OND: Operation New Dawn
OPC: Outpatient file
OR: Odds ratio
PBM: Pharmacy benefits management (database)
PCL-M: PTSD checklist – military
PI: Primary investigator
POSSE: Perioperative outcomes and safety in the seriously mentally ill elderly
POTIMP: Post-surgical outcomes for traumatically injured military patients
PSAS: Prosthetic and sensory aids service
PTF: Inpatient file
PTSD: Post-traumatic stress disorder
REALSSN: Real social security number
SAMMC: San Antonio Military Medical Center
SAS: Statistical analysis software
SCRSSN: Scrambled social security number
SDR: Service directed research
SF-36: Short form 36 (questions) health survey
SMFA: Short musculoskeletal function assessment
SQL: Structured Query Language
SSN: Social security number
STA: Station
STVHCS: South Texas Veterans Health Care System
TBI: Traumatic brain injury
TMDS: The theatre medical data store [TMDS]
TRACC: Trajectories of resilience and comorbidity clusters
USAISR: US Army Institute for Surgical Research
USAMRMC: U.S. Army Medical Research and Materiel Command
VA: Veterans Administration
VACO: Veterans Administration Central Office
VAST: VHA site tracking databases
VAVIS: Veterans Administration vascular injury study
VETSNET: Veterans service network (Corporate Mini Master File)
VHA HSR&D IIR: Veterans Health Administration health services research & development investigator initiated research
VistA: Veterans health information systems and technology architecture
VITALS: Vital statistics
VR-36: Veterans Rand 36-item health survey
WISPR: Workforce investment streamlined reporting system.
